# Calculation of Mercury’s Effects on Neurodevelopment

**DOI:** 10.1289/ehp.1206033

**Published:** 2012-12-03

**Authors:** Philippe Grandjean, Celine Pichery, Martine Bellanger, Esben Budtz-Jørgensen

**Affiliations:** 1 Harvard School of Public Health, Boston, Massachusetts, E-mail: pgrand@hsph.harvard.edu; 2 School for Advanced Studies in Public Health (EHESP), Paris, France; 3 Department of Biostatistics, University of Copenhagen, Copenhagen, Denmark

Bellinger (2012) recently estimated the loss of cognitive function in terms of Full-Scale intelligence quotient (IQ) in children exposed to certain environmental chemicals. To ascertain pre-natal exposures of methyl-mercury (MeHg) in children, he used exposure data on mercury (Hg) concentrations in hair of U.S. women of childbearing age (16–49 years) from NHANES (National Health and Nutrition Examination Survey) 1999–2000 (McDowell et al. 2004). Bellinger applied a regression coefficient of –0.18 IQ points per microgram per gram increase in maternal hair as calculated by Axelrad et al. (2007). However, the results of Axelrad et al. (2007) relied on incomplete data from a prospective study in the Faroe Islands and on non-adjusted results from the Seychelles study, later found to be confounded by nutrients from seafood (Strain et al. 2008). Bellinger (2012) then applied the regression coefficient to hair Hg levels > 1.11 µg/g (90th percentile), because this level corresponds to the reference dose of MeHg established many years ago. Assuming a concentration of 1.73 µg/g (95th percentile) as the midpoint (rather than the average, which is higher) for the hair Hg levels of the 10% of U.S. women with a level > 1.11 µg/g, he estimated a total IQ loss of 284,580 points. We believe that Bellinger’s general approach is sound but that the dose–response information is outdated, a caveat that Bellinger noted, although it was not reflected in the summary table. We therefore wish to complement these calculations using updated dose–response data.

Prospective data justify a lower threshold Hg level of 0.58 µg/g hair corresponding to 50% of the reference dose (Grandjean and Budtz-Jørgensen 2007). In addition, a 1-µg/g increase in hair Hg concentration is more likely associated with an average adverse impact of 0.465 IQ points, as discussed by Pichery et al. (2012). Assuming a log- normal exposure distribution, a 75th percentile hair Hg concentration of 0.42 µg/g, and a 90th percentile of 1.11 µg/g as reported by McDowell et al. (2004), we estimate that 18.5% of women exceed a threshold of 0.58 µg/g hair Hg and that the average concentration for 0.58–1.11 µg/g is approximately 0.8 µg/g. For the sake of comparing these values with Bellinger’s calculations (Bellinger 2012), we used a median concentration of 1.73 µg/g as the average hair Hg level of the 10% of U.S. women with a level > 1.11 µg/g. On the basis of these assumptions, we calculated a total IQ loss for the U.S. population of children 0–5 years of age (*n* = 25.5 million) to be 1,590,000 IQ points, or 264,000 IQ points per year.

We recently used similar calculations to estimate the annual costs of Hg pollution in France (Pichery et al. 2012), a country one-fifth the size of the United States. At slightly higher exposure levels, the annual loss in IQ points was estimated to be 157,000. Greater losses were obtained using a log-scale effect (Pichery et al. 2012). With an estimated value of each IQ point of $18,000 in terms of life-time earnings, the current loss of IQ points associated with MeHg exposure represents a very substantial value to society.
